# Predictive Coding with Neural Transmission Delays: A Real-Time Temporal Alignment Hypothesis

**DOI:** 10.1523/ENEURO.0412-18.2019

**Published:** 2019-05-06

**Authors:** Hinze Hogendoorn, Anthony N. Burkitt

**Affiliations:** 1 Melbourne School of Psychological Sciences, University of Melbourne, Melbourne, Victoria 3010, Australia; 2 Helmholtz Institute, Department of Experimental Psychology, Utrecht University, 3512 JE, Utrecht, The Netherlands; 3 NeuroEngineering Laboratory, Department of Biomedical Engineering, University of Melbourne, Melbourne, Victoria 3010, Australia

**Keywords:** alignment, extrapolation, neural delays, prediction, predictive coding, temporal

## Abstract

Hierarchical predictive coding is an influential model of cortical organization, in which sequential hierarchical levels are connected by backward connections carrying predictions, as well as forward connections carrying prediction errors. To date, however, predictive coding models have largely neglected to take into account that neural transmission itself takes time. For a time-varying stimulus, such as a moving object, this means that backward predictions become misaligned with new sensory input. We present an extended model implementing both forward and backward extrapolation mechanisms that realigns backward predictions to minimize prediction error. This realignment has the consequence that neural representations across all hierarchical levels become aligned in real time. Using visual motion as an example, we show that the model is neurally plausible, that it is consistent with evidence of extrapolation mechanisms throughout the visual hierarchy, that it predicts several known motion–position illusions in human observers, and that it provides a solution to the temporal binding problem.

## Visual Abstract

**Figure:**
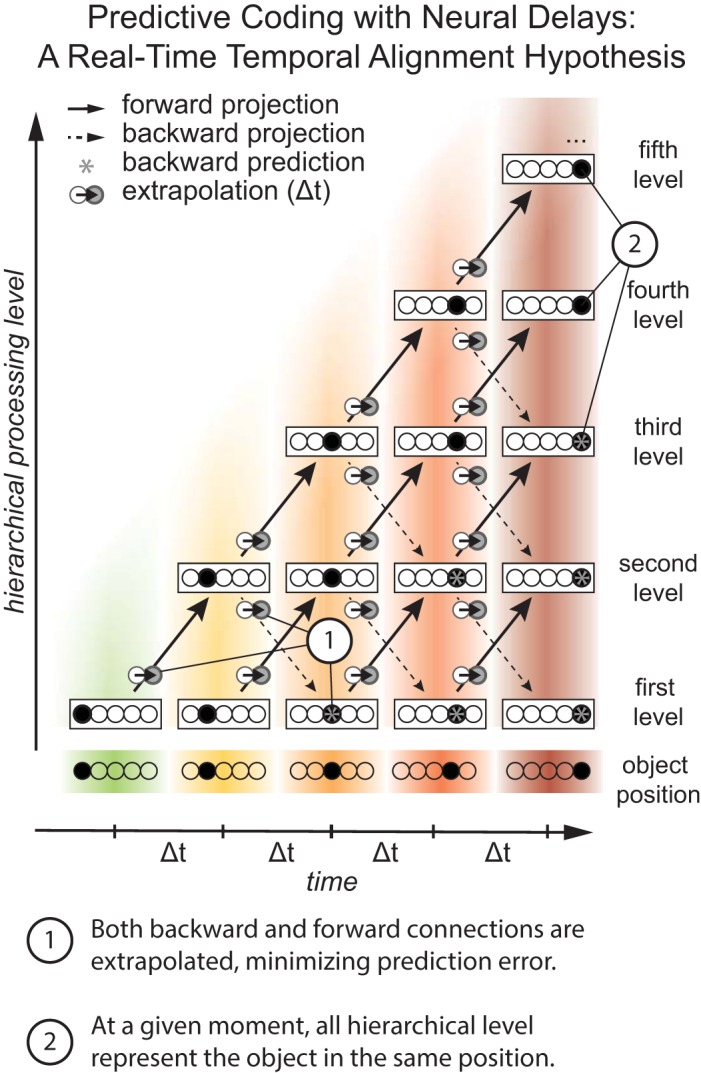

## Significance Statement

Despite the enormous scientific interest in predictive coding as a model of cortical processing, most models of predictive coding do not take into account that neural processing itself takes time. We show that when the framework is extended to allow for neural delays, a model emerges that provides a natural, parsimonious explanation for a wide range of experimental findings. It is consistent with neurophysiological data from animals, predicts a range of well known visual illusions in humans, and provides a principled solution to the temporal binding problem. Altogether, it explains how predictive coding mechanisms cause different brain areas to align in time despite their different delays, and in so doing it explains how cortical hierarchies function in real time.

## Introduction

Predictive coding is a model of neural organization that originates from a long history of proposals that the brain infers, or predicts, about the state of the world on the basis of sensory input (von Helmholtz, 1867; Gregory, 1980; Dayan et al., 1995). It has been particularly influential in the domain of visual perception (Srinivasan et al., 1982; Mumford, 1992; Rao and Ballard, 1999; Spratling, 2008, 2012), but has also been extensively applied in audition (for review, see Garrido et al., 2009; Bendixen et al., 2012), the somatosensory system (van Ede et al., 2011), motor control (Blakemore et al., 1998; Adams et al., 2013), and decision science (Schultz, 1998; Summerfield and de Lange, 2014), where it accounts for a range of subtle response properties and accords with physiology and neuroanatomy. Although it has been criticized for being insufficiently articulated (Kogo and Trengove, 2015), it has been developed further into a general theory of cortical organization (Bastos et al., 2012; Spratling, 2017) and even been advocated as a realization of the free-energy principle, a principle that might apply to all self-organizing biological systems (Friston, 2005, 2010, 2018; Friston and Kiebel, 2009).

**Figure 1.**
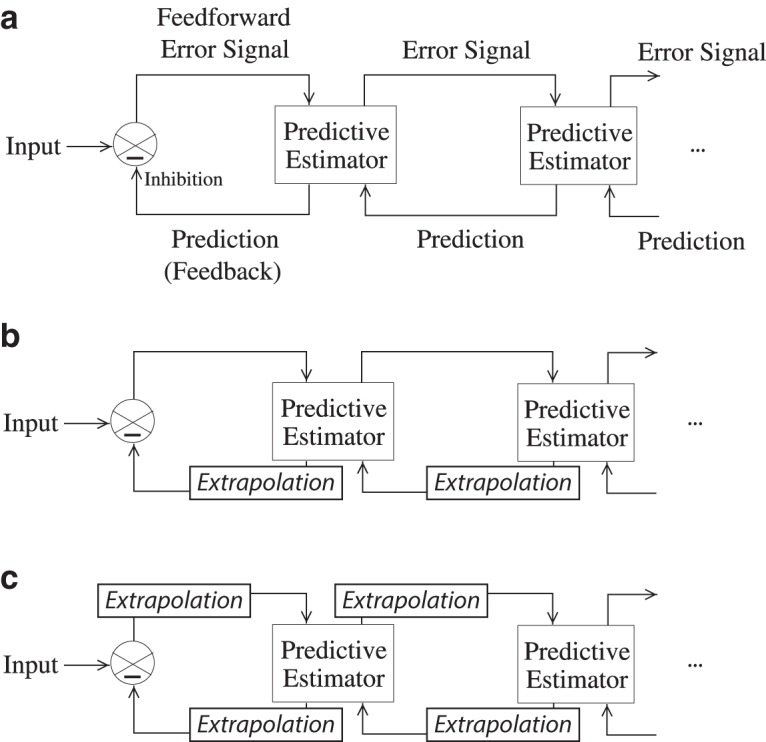
The classical hierarchical predictive coding model and two possible extensions. ***a***, The classical predictive model (model A). This model consists of hierarchically organized loops of forward and backward connections. Backward signals carry predictions, and forward signals carry prediction errors (Rao and Ballard, 1999). ***b***, The predictive model with extrapolated feedback (model B). To handle time-varying stimuli such as motion, the classical model can be expanded to include an extrapolation mechanism on the backward step. This would be one way to minimize prediction error for time-varying stimuli. ***c***, The predictive model with real-time alignment (model C). In this model, extrapolation mechanisms work on both forward and backward steps. Like model B, this would minimize prediction error, but has the additional consequence that it aligns the content of neural representations across the hierarchy. Diagram labels in ***b*** and ***c*** are as in ***a***, but are omitted for clarity.

An essential principle of predictive coding is a functional organization in which higher organizational units “predict” the activation of lower units. Those lower units then compare their afferent input to this backward prediction, and feed forward the difference: a prediction error (Rao and Ballard, 1999; where the term “prediction” is used in the strictly hierarchical sense, rather than the everyday temporal sense of predicting the future). This interaction of backward predictions and forward prediction errors characterizes each subsequent level of the processing hierarchy (Fig. 1*a*).

In this review, we argue that neural transmission delays cause the classical model of hierarchical predictive coding (Rao and Ballard, 1999) to break down when input to the hierarchy changes on a timescale comparable with that of the neural processing. Using visual motion as an example, we present two models that extend the classical model with extrapolation mechanisms to minimize prediction error for time-varying sensory input. One of these models, in which extrapolation mechanisms operate on both forward and back mechanisms, has as a consequence that all levels in the hierarchy become aligned in real time. We argue that this model not only minimizes prediction error, but also parsimoniously explains a range of spatiotemporal phenomena, including motion-induced position shifts such as the flash-lag effect and related illusions, and provides a natural solution to the question of how asynchronous processing of visual features nevertheless leads to a synchronous conscious experience (the temporal binding problem).

### Minimizing prediction error

The core principle behind the pattern of functional connectivity in hierarchical predictive coding is the minimization of total prediction error. This is considered to be the driving principle on multiple biological timescales (Friston, 2018).

At long timescales, the minimization of prediction error drives evolution and phylogenesis. Neural signaling is metabolically expensive, and there is therefore evolutionary pressure for an organism to evolve a system of neural representation that allows for complex patterns of information to be represented with minimal neural firing. A sparse, higher-order representation that inhibits the costly, redundant activity of lower levels would provide a metabolic, and therefore evolutionary, advantage. Theoretically, the imperative to minimize complexity and metabolic cost is an inherent part of free-energy (i.e., prediction error) minimization. This follows because the free energy provides a proxy for model evidence, and model evidence is accuracy minus complexity (Friston, 2010). This means that minimizing free energy is effectively the same as self-evidencing (Hohwy, 2016), and both entail a minimization of complexity or computational costs, and their thermodynamic concomitants.

At the level of individual organisms, at timescales relevant to decision-making and behavior, the minimization of prediction error drives learning (den Ouden et al., 2012; Friston, 2018). Predictions are made on the basis of an internal model of the world, with the brain essentially using sensory input to predict the underlying causes of that input. The better this internal model fits the world, the better the prediction that can be made, and the lower the prediction error. Minimizing prediction error therefore drives a neural circuit to improve its representation of the world: in other words, learning.

Finally, at subsecond timescales relevant to sensory processing, the minimization of prediction error drives the generation of stable perceptual representations. A given pattern of sensory input feeds in to forward and backward mechanisms that iteratively projects to each other until a dynamic equilibrium is reached between higher-order predictions and (local) deviations from those predictions. Because this equilibrium is the most efficient representation of the incoming sensory input, the principle of prediction error minimization works to maintain this representation as long as the stimulus is present. This results in a perceptual representation that remains stable over time. Interestingly, because there may be local minima in the function determining total prediction error, a given stimulus might have multiple stable interpretations, as is, for example, the case for ambiguous stimuli such as the famous Necker cube (Necker, 1832).

### Hierarchical versus temporal prediction

In discussing predictive coding, there is an important distinction to be made regarding the sense in which predictive coding models predict.

Descriptively, predictive coding models are typically considered (either implicitly or explicitly) to reflect some kind of expectation about the future. For example, in perception, preactivation of the neural representation of an expected sensory event ahead of the actual occurrence of that event reflects the nervous system predicting a future event (Garrido et al., 2009; Kok et al., 2017; Hogendoorn and Burkitt, 2018). In a decision-making context, a prediction might likewise be a belief about the future consequences of a particular choice (Sterzer et al., 2018). These are predictions in the temporal sense of predicting future patterns of neural activity.

However, conventional models of predictive coding such as the one first proposed by Rao and Ballard (1999) are not predictive in this same sense. Rather than predicting the future, these models are predictive in the hierarchical sense of higher areas predicting the activity of lower areas (Rao and Ballard, 1999; Bastos et al., 2012; Spratling, 2012, 2017). These models do not predict what is going to happen: rather, by converging on a configuration of neural activity that optimizes the representation of a stable sensory input, they hierarchically predict what is happening. The use of the word “prediction” in this context is therefore somewhat unfortunate, as conventional models of predictive coding do not actually present a mechanism that predicts in the temporal way that prediction is typically used in ordinary discourse.

### Predicting the future

To date, the temporal dimension has been nearly absent from computational work on predictive coding. With the exception of generalized formulations (see below), it is generally implicitly assumed that sensory input is a stationary process (i.e., that it remains unchanged until the system converges on a minimal prediction error solution). The available studies of dynamic stimuli in a predictive coding context consider only autocorrelations within the time series of a given neuron: in other words, the tendency of sensory input to remain the same from moment to moment (van Hateren and Ruderman, 1998; Rao, 1999; Huang and Rao, 2011). Computationally, this prediction is easily implemented as a biphasic temporal impulse response function (Dong and Atick, 1995; Dan et al., 1996; Huang and Rao, 2011), which is consistent with the known properties of neurons in the early visual pathway, including the lateral geniculate nucleus (LGN; Dong and Atick, 1995; Dan et al., 1996). However, this is only the most minimal temporal prediction that a neural system might make: the prediction that its input remains unchanged over time.

A more general framework is provided by generalized (Bayesian) filtering (Friston et al., 2010). The key aspect of generalized predictive coding is that instead of just predicting the current state of sensory impressions, there is an explicit representation of velocity, acceleration, and other higher orders of motion. This sort of representation has been used to model oculomotor delays during active vision (Perrinet et al., 2014). However, neither this framework nor predictive coding approaches implicit in Kalman filters (that include a velocity-based prediction) explicitly account for transmission delays.

As such, what is missing from conventional models of predictive coding is the fact that neural communication itself takes time. Perhaps because the delays involved in *in silico* simulations are negligible, or perhaps because the model was first articulated for stationary processes, namely static images and stable neural representations, models of neural circuitry have entirely neglected to take into account that neural transmission incurs significant delays. These delays mean that forward and backward signals are misaligned in time. For an event at a given moment, the sensory representation at the first level of the hierarchy needs to be fed forward to the next hierarchical level, where a prediction is formulated, which is then fed back to the original level. In the case of a dynamic stimulus, however, by the time that the prediction arrives back at the first hierarchical level, the stimulus will have changed, and the first level will be representing the new state of that stimulus. As a result, backward predictions will be compared against more recent sensory information than the information on which they were originally based, and which they were sent to suppress. If this temporal misalignment between forward and backward signals would not somehow be compensated for, under the classical hierarchical predictive coding model any time-varying stimulus would generate very large prediction errors at each level of representation, which is typically not seen in electrophysiological recordings of responses to stimuli with constant motion (as opposed to unexpected changes in the trajectory; Schwartz et al., 2007; McMillan and Gray, 2012; Dick and Gray, 2014).

Because of neural transmission delays, prediction error is minimized not when a backward signal represents the sensory information that originally generated it, but when it represents the sensory information that is going to be available at the lower level by the time the backward signal arrives. In other words, prediction error is minimized when the backward signal anticipates the future state of the lower hierarchical level. For stimuli that are changing at a constant rate, estimating that future state requires only rate-of-change information about the relevant feature, and it follows that if such information is available at a given level, it will be recruited to minimize prediction error. When allowing for transmission delays, hierarchical predictions therefore need to become temporal predictions: they need to predict the future.

### Two extended models

A clear example of common, time-varying sensory input is visual motion. Here, we use visual motion to illustrate the limitations of the classical predictive coding model when input is time varying, and present two extensions to the classical model that would solve these limitations. In this illustration, we consider neural populations at various levels of the visual hierarchy that represent position, for instance as a Gaussian population code. Additionally, we will argue that to effectively represent position despite transmission delays, those neural populations will additionally represent velocity. Consequently, each neural population representing a particular position would consist of subpopulations representing the range of stimulus velocities.

#### The classical predictive model (model A)

**Figure 2.**
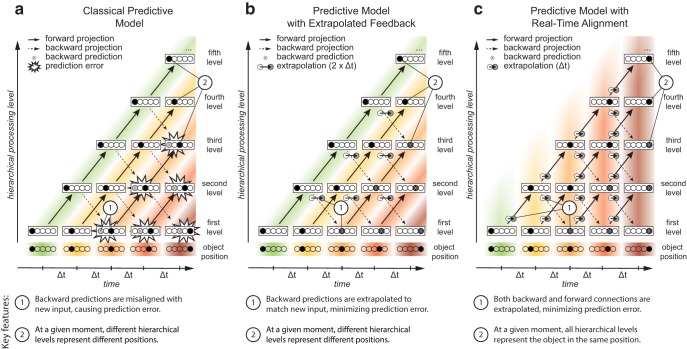
Three simplified models of the representation of the position of a moving object throughout the visual processing hierarchy under the predictive coding framework. Each rectangle denotes the neural representation of the position of the object at a given hierarchical level and at a given time, with the filled circle indicating the object in one of five possible positions. In this simplified representation, all connections are modeled as incurring an equal transmission delay (Δt). Colored bands link corresponding representations, and numbered circles highlight the core features of each model. ***a***, The classical predictive model (model A) of predictive coding comprises forward connections from one hierarchical level to the next level (solid lines), and backward connections to the previous level (dashed lines). No allowance is made for neural transmission delays, such that backward connections carry a position representation (asterisks) that is outdated by the time that signal arrives. The resulting mismatch with more recent sensory input generates large errors, which subsequently propagate through the hierarchy (emphasized with starbursts). ***b***, In the predictive model with extrapolated feedback (model B), an extrapolation mechanism operates on predictive backward projections, anticipating the future position of the object. This mechanism on the backward projections compensates for the total time-cost incurred during both the forward and the backward portion of the loop. This would minimize total prediction error in this simplified model. However, the mechanism would rapidly become more complex when one considers that individual areas tend to send and receive signals to and from multiple levels of the hierarchy. ***c***, In the predictive model with real-time alignment (model C), extrapolation mechanisms compensate for neural delays at both forward and backward steps. This parsimoniously minimizes total prediction error, even for more complex connectivity patterns. Additionally, the model differs from the first two models in that at any given time, all hierarchical levels represent the same position. Conversely, in the first two models, at any given time neural transmission delays mean that all hierarchical levels represent a different position. This crucial difference is evident as vertical, rather than diagonal, colored bands linking matching representations across the hierarchy. The consequence of this hypothesis is therefore that the entire visual hierarchy becomes temporally aligned. This provides an automatic and elegant solution to the computational challenge of establishing which neural signals belong together in time: the temporal binding problem. It is also consistent with demonstrated extrapolation mechanisms in forward pathways and provides a parsimonious explanation for a range of motion-induced position shifts.

In the classical hierarchical predictive coding model (Rao and Ballard, 1999; Fig. 1*a*), neural transmission delays mean that backward predictions arrive at a level in the processing pathway a significant interval of time after that level fed forward the signal that led to those predictions. Because the input to this level has changed during the elapsed time, this results in prediction error (Fig. 2*a*).

#### The predictive model with extrapolated feedback (model B)

In this extension to the classical model, each complete forward/backward loop uses any available rate-of-change information to minimize prediction error. In the case of visual motion, this means that the circuit will use concurrent velocity information to anticipate the representation at the lower level by the time the backward signal arrives at that level. In this model, an extrapolation mechanism is implemented only at the backward step of each loop (Fig. 1*b*) to minimize predictive error while leaving the forward sweep of information unchanged (Fig. 2*b*).

#### The predictive model with real-time alignment (model C)

In the classical model, prediction error results from the cumulative delay of both forward and backward transmission. Prediction error is minimized when this cumulative delay is compensated at any point in the forward–backward loop. Evidence from both perception (Nijhawan, 1994, 2008) and action (Soechting et al., 2009; Zago et al., 2009) strongly suggests that at least part of the total delay incurred is compensated by extrapolation at the forward step. Accordingly, in this model, we propose that extrapolation mechanisms work on both forward and backward signals: any signal that is sent from one level to another, whether forward or backward, is extrapolated into the future that precisely compensates for the delay incurred by that signal while it is in transit (Fig. 1*c*). In addition to minimizing prediction error, this model has the remarkable consequence of synchronizing representations throughout the hierarchy: under this model, all levels represent a moving object in the same position at the same moment (Fig. 2*c*), independent of where in the hierarchy each level lies.

### Evaluating the evidence

We have argued above that the classical model of predictive coding (Rao and Ballard, 1999; Huang and Rao, 2011; Bastos et al., 2012; Spratling, 2017) will consistently produce prediction errors when stimuli are time varying and neural transmission delays are taken into consideration (Fig. 2*a*). We have proposed two possible extensions to the classical model, each of which would minimize prediction error. Here, we evaluate the evidence for and against each of these two models.

#### Neural plausibility

Models B and C are both neurally plausible. First, both models share key features with existing computational models of motion–position interaction. For example, Kwon et al. (2015) recently advanced an elegant computational model uniting motion and position perception. An interaction between motion and position signals is a central premise of their model, such that instantaneous position judgments are biased by concurrent motion stimuli in a way that is qualitatively similar to the model we propose here. In fact, they demonstrate that this interaction predicts a number of properties of a visual effect known as the curveball illusion, in which the internal motion of an object causes its position (and trajectory) to be misperceived. Below, we argue that our extension of hierarchical predictive coding similarly predicts a number of other motion-induced position shifts. One such illusion is the flash-lag effect (Nijhawan, 1994), in which a stationary bar is flashed in alignment with a moving bar, but is perceived to lag behind it. Importantly, Khoei et al. (2017) recently presented a detailed argument that the flash-lag effect could be explained by a Bayesian integration of motion and position signals, and postulated that such mechanisms might compensate for neural delays. This interpretation of the interaction between motion and position signals is entirely consistent with the mechanisms we propose in models B and C. Finally, we have previously argued that the prediction errors that would necessarily arise under these models when objects unexpectedly change direction would lead to another perceptual effect known as the flash-grab illusion (Cavanagh and Anstis, 2013; van Heusden et al., 2019; Fig. 1). Consequently, the models we propose are compatible with existing empirical and computational work.

Second, both extensions to the classical model incorporate neural extrapolation mechanisms at each level of the visual hierarchy. This requires first that information about the rate of change be represented and available at each level. In the example of visual motion, it is well established that velocity is explicitly represented throughout the early visual hierarchy, including the retina (Barlow and Levick, 1965), LGN (Marshel et al., 2012; Cheong et al., 2013; Cruz-Martín et al., 2014; Zaltsman et al., 2015), and primary visual cortex (V1; Hubel and Wiesel, 1968). As required by both models, velocity information is therefore available throughout the hierarchy. Indeed, it follows from the prediction error minimization principle that if velocity is available at each level, it can and will be used at each level to optimize the accuracy of backward predictions involving that level.

Finally, the extrapolation processes posited in both models should cause activity ahead of the position of a moving object, preactivating (i.e., priming the neural representation of) the area of space into which the object is expected to move. This activation ahead of a moving object is consistent with the reported preactivation of predicted stimulus position in cat (Jancke et al., 2004) and macaque (Subramaniyan et al., 2018) V1, as well as in human EEG (Hogendoorn and Burkitt, 2018). Importantly, if the object unexpectedly vanishes, such extrapolation would preactivate areas of space in which the object ultimately never appeared. This is consistent with psychophysical experiments where an object is perceived to move into areas of the retina where no actual stimulus energy can be detected (Maus and Nijhawan, 2008; Shi and Nijhawan, 2012), as well as with recent fMRI work showing activation in retinotopic areas of the visual field beyond the end of the motion trajectory (Schellekens et al., 2016). Although the models presented here are by no means the only models that would be able to explain these results, these results do demonstrate properties predicted by models B and C.

#### Hierarchical complexity

Model C is more robust to hierarchical complexity than model B. Figure 2 shows extremely simplified hierarchies: it omits connections that span multiple hierarchical levels, it represents all time delays as equal and constant, and it shows only a single projection from each area. In reality, of course, any given area is connected to numerous other areas (Felleman and Van Essen, 1991), each with different receptive field properties. Transmission delays will inevitably differ depending on where forward signals are sent to and where backward signals originate from. Importantly, a given area might receive input about the same moment through different pathways with different lags. A purely backward extrapolation process (model B) would not naturally compensate for these different lags, because it would not be able to differentiate between the forward signals that led to the prediction. Conversely, a model with extrapolation mechanisms at both forward and backward connections (model C) would be able to compensate for any degree of hierarchical complexity: for a feedback loop with a large transmission delay, prediction error would be minimized by extrapolating further along a trajectory than for a feedback loop with a smaller transmission delay. In this way, each connection would essentially compensate for its own delay.

#### Motion-induced position shifts

Model C predicts motion-induced position shifts, namely visual illusions in which motion signals affect the perceived positions of objects. In model C, the representation in higher hierarchical levels does not represent the properties of the stimulus as it was when it was initially detected, but rather the expected properties of that stimulus at a certain extent into the future. In the case of visual motion, this means that the forward signal represents the extrapolated position rather than the detected position of the moving object. One consequence is that at higher hierarchical levels, a moving object is never represented in the position at which it first appears (Fig. 2*c*, top diagonal). Instead, it is first represented at a certain extent along its (expected) trajectory. Model C therefore neatly predicts a visual phenomenon known as the Fröhlich effect, in which the initial position of an object that suddenly appears in motion is perceived as being shifted in the direction of its motion (Fröhlich, 1924; Kirschfeld and Kammer, 1999). Conversely, model B cannot explain these phenomena, as the feedforward position representation reflects the veridical location of the stimulus, rather than its extrapolated position (Fig. 2*b*).

Model C is also consistent with the much studied flash-lag phenomenon (Nijhawan, 1994), in which a briefly flashed stimulus that is presented in alignment with a moving stimulus appears to lag behind that stimulus. Although alternative explanations have been proposed (Eagleman and Sejnowski, 2000), a prominent interpretation of this effect is that it reflects motion extrapolation, specifically implemented to compensate for neural delays (Nijhawan, 1994, 2002, 2008). Model C is not only compatible with that interpretation, but it provides a principled argument (prediction error minimization) for why such mechanisms might develop.

#### Neural computations

There are two key computations that the neural-processing hierarchy needs to carry out to implement the proposed real-time alignment of model C. First, it is necessary that the position representation at each successive level incorporates the effect of the neural transmission delay to compensate for the motion, rather than simply reproducing the position representation at the preceding level. Second, this delay-and-motion-dependent shift of the position representation must occur on both the forward and backward connections in the hierarchy. Moreover, these computations must be learned through a process that is plausible in terms of synaptic plasticity, namely that the change in a synaptic strength is activity dependent and local. The locality constraint of synaptic plasticity requires that changes in the synaptic strength (i.e., the weight of a connection) can only depend on the activity of the presynaptic neuron and the postsynaptic neuron. Consequently, the spatial distribution of the synaptic changes in response to a stimulus are confined to the spatial distribution of the position representation of the stimulus, which has important consequences for the structure of the network that emerges as a result of learning.

By incorporating the velocity of the stimuli into the prediction error minimization principle, it becomes possible for a learning process to selectively potentiate the synapses that lead to the hierarchically organized position representations in both models B and C. This requires that the appropriate velocity subpopulation of each position representation is activated to generate the neural representations illustrated in Figure 2, *b* and *c*. The prediction error is generated by the extent of misalignment of the actual input and the predicted input position representation on the feedback path, which involves the combined forward and backward paths, as illustrated in Figure 2. Consequently, the prediction error minimization principle results in changes to the weights on both the forward and backward paths. What distinguishes models B and C is that the probability of potentiation depends on the extent of the spatial overlap of the position representations at successive times. In model C, this spatial overlap between the forward and backward paths is the same, namely the position representation has changed by the same extent during both the forward and backward transmission delays, assuming that the neural delay time is the same for both paths. However, in model B the forward and backward paths are quite different: the forward path has complete congruence in the position representation between adjacent levels, whereas the backward path has a position representation that corresponds to the sum of the forward and backward delays, so that the position representations between the two levels on the backward path are much further apart. Since the position representations are local with a distribution that falls off from the center (e.g., an exponential or power-law fall off), the probability of potentiation of the backward pathway in model B is correspondingly lower (by an exponential or power-law factor). As a result, a local learning rule that implements the prediction error principle would tend to favor the more equal distribution of delays between the forward and backward paths of model C. Since the same learning principle applies to weights between each successive level of the hierarchical structure, it is capable of providing the basis for the emergence of this structure during development.

Both models B and C posit interactions between motion and position signals at multiple levels in the hierarchy. This is compatible with a number of theoretical and computational models of motion and position perception. For example, Eagleman and Sejnowski (2007) have argued that for a whole class of motion-induced position shifts, a local motion signal biases perceived position, precisely the local interactions between velocity and position signals that we propose. Furthermore, these instantaneous velocity signals have been shown to affect not only the perceptual localization of concurrently presented targets, but also the planning of saccadic eye movements aimed at those targets (Quinet and Goffart, 2015; van Heusden et al., 2018). We argue that, under the hierarchical predictive coding framework, delays in neural processing necessarily lead to the evolution of motion–position interactions.

Furthermore, both models B and C posit such interactions at multiple levels, including very early levels in the hierarchy. This is consistent with recent work on the flash-grab effect, a motion-induced position shift in which a target briefly flashed on a moving background that reverses direction is perceived to be shifted away from its true position (Cavanagh and Anstis, 2013). Using EEG, the interaction between motion and position signals that generates the illusion has been shown to have already occurred within the first 80 ms following stimulus presentation, indicating a very early locus of interaction. A follow-up study using dichoptic presentation revealed that, even within this narrow time frame, extrapolation took place in at least two dissociable processing levels (van Heusden et al., 2019).

#### Temporal alignment

A defining feature of model C is that, due to extrapolation at each forward step, all hierarchical areas become aligned in time. Although neural transmission delays mean that it takes successively longer for a new stimulus to be represented at successive levels of the hierarchy (Lamme et al., 1998), the fact that the neural signal is extrapolated into the future at each level means that the representational content of each consecutive level now runs aligned with the first hierarchical level, and potentially with the world, in real time. In the case of motion, we perceive moving objects where they are, rather than where they were, because the visual system extrapolates the position of those objects to compensate for the delays incurred during processing. Of course, the proposal that the brain compensates neural delays by extrapolation is not new (for review, see Nijhawan, 2008). Rather, what is new here is the recognition that this mechanism has not developed for the purpose of compensating for delays at the behavioral level, but that it follows necessarily from the fundamental principles of predictive coding.

The temporal alignment characterizing model C also provides a natural solution to the problem of temporal binding. Different visual features (e.g., contours, color, motion) are processed in different, specialized brain areas (Livingstone and Hubel, 1988; Felleman and Van Essen, 1991). Due to anatomic and physiologic differences, these areas process their information with different latencies, which leads to asynchrony in visual processing. For example, the extraction of color has been argued to lead the extraction of motion (Moutoussis and Zeki, 1997b; Zeki and Bartels, 1998; Arnold et al., 2001). The question therefore arises of how these asynchronously processed features are nevertheless experienced as a coherent stream of visual awareness, with components of that experience processed in different places at different times (Zeki and Bartels, 1999). Model C intrinsically solves this problem. Because the representation in each area is extrapolated to compensate for the delay incurred in connecting to that area, representations across areas (and therefore features) become aligned in time.

#### Local time perception

Model C eliminates the need for a central timing mechanism. Because temporal alignment in this model is an automatic consequence of prediction error minimization at the level of local circuits, no central timing mechanism (e.g., internal clock; Gibbon, 1977) is required to carry out this alignment. Indeed, under this model, if a temporal property of the prediction loop changed for a particular part of the visual field, then this would be expected to result in localized changes in temporal alignment. Although this is at odds with our intuitive experience of the unified passage of time, local adaptation to flicker has been found to distort time perception in a specific location in the visual field (Johnston et al., 2006; Hogendoorn et al., 2010). Indeed, these spatially localized distortions have been argued to result from adaptive changes to the temporal impulse response function in the LGN (Johnston, 2010, 2014), which would disrupt the calibration of any neuron extrapolating a given amount of time into the future. By pointing to adaptation in specific LGN populations, this model has emphasized the importance of local circuitry in explaining the spatially localized nature of this temporal illusion. The counterintuitive empirical finding that the perceived timing of events in specific positions in the visual field can be distorted by local adaptation is therefore consistent with the local compensation mechanisms that form part of model C.

#### Violated predictions

Under the assumption that the observer’s percept corresponds to the current stable representation of the stimulus at a given level of representation, models B and C produce stable percepts when objects move on predictable trajectories. In model B, the represented location at any given moment will lag behind the outside world by an amount dependent on the accumulated delay in the hierarchy, and in model C the represented location will be synchronous with the outside world. In both cases, however, the representation at the appropriate level, and hence the conscious percept, will continue to stably evolve. In contrast, when events do not unfold predictably, such as when objects unexpectedly appear, disappear, or change their trajectory, this introduces new forward information, which is, of course, at odds with backward predictions at each level. This gives rise to prediction errors at each level of the hierarchy, with progressively larger prediction errors further up the hierarchy (as it takes longer for sensory information signaling a violation of the status quo to arrive at higher levels). During the intervening time, those areas will continue to (erroneously) extrapolate into a future that never comes to pass.

The breakdown of prediction error minimization (and therefore accurate perception) in situations where events unfold unpredictably fits with empirical studies showing visual illusions in these situations. As noted above, the flash-lag effect is one such visual illusion. Under model C, this effect occurs because the position of the moving object can be extrapolated due to its predictability, whereas the flashed object, which is unpredictable, cannot (Nijhawan, 1994). As the sensory information pertaining to the position of the moving object ascends the visual hierarchy, it is extrapolated at each level. Conversely, the representation of the flash is passed on “as-is,” such that a mismatch accumulates between the two. This mismatch between the predictable motion and the unpredictable flash has a parallel in the flash-grab effect, a visual illusion whereby a flash is presented on a moving background that reverses direction (Cavanagh and Anstis, 2013). The result is that the perceived position of the flash is shifted away from the first motion sequence (Blom et al., 2019) and in the direction of the second motion sequence. Importantly, a recent study parametrically varied the predictability of the flash and showed that the strength of the resulting illusion decreased as the flash became more predictable (Adamian and Cavanagh, 2016). Likewise, in studies of temporal binding, the asynchrony in the processing of color and motion is evident only when rapidly moving stimuli abruptly change direction (Moutoussis and Zeki, 1997a). These illusions reveal that accurate perception breaks down when prediction is not possible, consistent with model C, but not with the classical predictive model A.

### Conclusions and future directions

Altogether, multiple lines of evidence converge in support of model C: an extension of the hierarchical predictive coding framework in which extrapolation mechanisms work on both forward and backward connections to minimize prediction error. In this model, minimal prediction error is achieved by local extrapolation mechanisms compensating for the specific delays incurred by individual connections at each level of the processing hierarchy. As a result, neural representations across the hierarchy become aligned in real time. This model provides an extension to classical predictive coding models that is necessary to account for neural transmission delays. In addition, the model predicts and explains a wide range of spatiotemporal phenomena, including motion-induced position shifts, the temporal binding problem, and localized distortions of perceived time.

#### Neural implementation

We have taken error minimization as the organizing principle of predictive coding, but now extended to incorporate the local velocity of the stimuli. This is in keeping with the approach of previous authors, who have successfully modeled hierarchical prediction error minimization (Rao and Ballard, 1997, 1999; Friston, 2010; Huang and Rao, 2011; Bastos et al., 2012; Spratling, 2017) and motion–position interactions (Kwon et al., 2015) as a Kalman filter (Kalman, 1960). The extension proposed here can, in principle, be implemented straightforwardly by incorporating the local velocity as an additional state variable in a manner analogous to that proposed for motion–position interactions (Kwon et al., 2015). Such an approach, in which the prediction error minimization incorporates the expected changes that occur due to both the motion of the stimulus and the propagation of the neural signals, is possible when the local velocity is one of the state variables that each level of the hierarchy has access to. This could be seen as a special case of generalized predictive coding, where there is an explicit leveraging of (sometimes higher-order) motion representations (Friston et al., 2010). This framework even goes beyond the first-order velocity implicit in a Kalman filter to include higher orders of motion. Mathematically, this makes it easy to extrapolate forward and backward in time by simply taking linear mixtures of different higher order motion signals using Taylor expansions (Perrinet et al., 2014). This would be one neurally plausible way in which the extrapolation in Figure 2 could be implemented.

Furthermore, it is important to note that by minimizing prediction error, the system will automatically self-organize as if it “knows” what neural transmission delays are incurred at each step. However, models B and C do not require these delays to be explicitly represented at all. Rather, if position and velocity are corepresented as state variables, and the system is exposed to time-variant input (e.g., a moving object), then selective Hebbian potentiation would suffice to strengthen those combinations of connections that cause the backward projection to accurately intercept the new sensory input. This would be one possible implementation of how extrapolation mechanisms calibrate to rate-of-change signals.

It is important to note that the proposed model extends, rather than replaces, the conventional formulation of predictive coding as first posited by Rao and Ballard (1999). We have not discussed how this model would function in the spatial domain to develop different receptive field properties at each level, as this has been discussed at length by other authors (Rao, 1999; Rao and Ballard, 1999; Jehee et al., 2006; Huang and Rao, 2011), and we intend our model to inherit these characteristics. Our model only extends the conventional model by providing for the situation when input is time variant. When it is not (i.e., for static stimuli), our model reduces to the conventional model.

Altogether, the details of how cortical circuits implement extrapolation processing, how those circuits interact with receptive field properties at different levels, and what synaptic plasticity mechanisms underlie the formation of these circuits still remain to be elaborated, and these are key areas for future research.

#### Prediction in the retina

The proposed model follows from the principle of error minimization within predictive feedback loops. These feedback loops are ubiquitous throughout the visual pathway, including backward connections from V1 to LGN. Although there are no backward connections to the retina, extrapolation mechanisms have nevertheless been reported in the retina (Berry et al., 1999), and these mechanisms have even been found to produce a specific response to reversals of motion direction, much akin to a prediction error (Holy, 2007; Schwartz et al., 2007; Chen et al., 2014). Indeed, the retina has been argued to implement its own, essentially self-contained predictive coding mechanisms that adaptively adjust spatiotemporal receptive fields (Hosoya et al., 2005). In the absence of backward connections to the retina, our model does not directly predict these mechanisms, instead predicting only extrapolation mechanisms in the rest of the visual pathway where backward connections are ubiquitous. On the long timescale, compensation for neural delays in the retina provides a behavioral, and therefore evolutionary, advantage, but more research will be necessary to address whether any short-term learning mechanisms play a role in the development of these circuits.

#### The ubiquity of velocity signals

We have emphasized hierarchical mechanisms at early levels of visual processing, consistent with extrapolation in monocular channels (van Heusden et al., 2019), early EEG correlates of extrapolation (Hogendoorn et al., 2015), and evidence that extrapolation mechanisms are shared for both perceptual localization and saccadic targeting (van Heusden et al., 2018). However, an influential body of literature has proposed that the human visual system is organized into two (partly) dissociable pathways: a ventral “what” pathway for object recognition and identification, and a dorsal “where” pathway for localization and motion perception (Mishkin and Ungerleider, 1982; Goodale et al., 1991; Goodale and Milner, 1992; Aglioti et al., 1995). Two decades later, the distinction is more nuanced (Milner and Goodale, 2008; Gilaie-Dotan, 2016), but the question remains whether velocity signals are truly ubiquitous throughout the visual hierarchy. However, given that the identity of a moving object typically changes much more slowly than its position, it may be that the neuronal machinery that underwrites (generalized) predictive coding in the ventral stream does not have to accommodate transmission delays. This may provide an interesting interpretation of the characteristic physiologic time constants associated with the magnocellular stream (directed toward the dorsal stream), compared with the parvocellular stream implicated in object recognition (Zeki and Shipp, 1988). Consequently, whether real-time temporal alignment is restricted to the “where” pathway or is a general feature of cortical processing that remains to be elucidated.

#### The functional role of prediction error

Throughout this perspective, we have considered prediction error as something that an effective predictive coding model should minimize. From the perspective of the free energy principle, the imperative to minimize prediction error can be argued from first principles. The sum of squared prediction error is effectively variational free energy, and any system that minimizes free energy will therefore self-organize to its preferred physiologic and perceptual states. However, we have not addressed the functional role of the prediction error signal itself. As noted by several authors, this signal might serve an alerting or surprise function (for review, see den Ouden et al., 2012). In the model proposed here, the signal might have the additional function of correcting a faulty extrapolation (Nijhawan, 2002, 2008; Shi and Nijhawan, 2012). In this role, prediction error signals would work to eliminate lingering neural traces of predictions that were unsubstantiated by sensory input: expected events that did not end up happening. This corrective function has been modeled as a “catch-up” effect for trajectory reversals in the retina (Holy, 2007; Schwartz et al., 2007), and as an increase in position uncertainty (and therefore an increase in relative reliance on sensory information) when objects change trajectory in a recently proposed Bayesian model of visual motion and position perception (Kwon et al., 2015). Further identifying the functional role of these signals is an exciting avenue for future research.

## Synthesis

Reviewing Editor: Li Li, New York University Shanghai Decisions are customarily a result of the Reviewing Editor and the peer reviewers coming together and discussing their recommendations until a consensus is reached. When revisions are invited, a fact-based synthesis statement explaining their decision and outlining what is needed to prepare a revision will be listed below. The following reviewer(s) agreed to reveal their identity: Karl Friston, Woon Ju Park.

Both reviewers think the ms addressed an important and interesting question in the field. However, both have concerns that have to be addressed before the ms can be considered for publication. Please pay special attention to Reviewer 2's complains about lack of explanation for the core concepts of the study. Their detailed comments are listed below.

Reviewer 1

I enjoyed reading this compelling and clearly described account of how generalised predictive coding could explain many phenomena in visual neuroscience and psychophysics. I think you did a very good job of unpacking the functional architectures implied by predictive coding models - and surveying the phenomena that can be explained. My slight concern is that you are unaware that the extrapolation and temporal alignment extensions already exist in generalised predictive coding. I think that you can nuance your introduction and discussion to resolve this as follows:

Line 105: at this point, you should to introduce the notion of generalised filtering/predictive coding. Perhaps with something like:

“... models of predictive coding with the exception of generalised formulations. Generalised predictive coding is, mathematically, synonymous with generalised (Bayesian) filtering (Friston, Stephan, Li, & Daunizeau, 2010). The key aspect of generalised predictive coding is that it works in generalised quadrants of motion. In other words, instead of just predicting the current state of sensory impressions, there is an explicit representation of velocity, acceleration and other higher orders of motion. This sort of representation has been used to model transmission delays during active vision - that are necessarily present in terms of oculomotor delays to and from the visual cortex and oculomotor system (Perrinet, Adams, & Friston, 2014). However, even vanilla predictive coding implicit in Kalman filters (that include a velocity-based prediction) do not explicitly account for transmission delays. In other words, it is implicitly assumed that...”

Line 115: could you replace phrases like “mechanistic models of predictive coding” and “classical” formulations of predictive coding with “conventional” - defining conventional predictive coding as a scheme based upon differential equations as opposed to time-delayed differential equations that incorporate transmission delays.

I thought figure 2 was excellent.

Line 355: perhaps you could include an explicit section on active vision; starting with something like:

“In active vision or inference, the opportunity to properly accommodate transmission delays becomes extremely prescient - given the oculomotor delays during eye movements can sometimes exceed 100 ms. This issue has received attention in theoretical neurobiology by the averaging generalised formulations of predictive coding. In this setting...

Line 373: could you say something like:

“...motion precision interactions. Technically speaking these are all special cases of generalised predictive coding, where there is an explicit leveraging of motion representations - sometimes high order. In other words, it is possible to go beyond the first order velocity implicit in a Kalman filter to include higher orders of motion. Mathematically, this means that it is easy to extrapolate forwards and backwards in time by simply taking linear mixtures of different higher order motion is using Taylor expansions - see (Perrinet et al., 2014). Neuronally, this represents a plausible way in which the extrapolation in Figure 2 could be implemented.”

Line 402: perhaps you could add:

“However, given that the identity of a moving object changes much more slowly than its position, it may be that the neuronal machinery that underwrites (generalised) predictive coding in the ventral stream does not have to accommodate transmission delays. This may provide an interesting interpretation of the characteristic physiological time constants associated with the magnocellular stream (directed towards the dorsal stream - as compared to the parvocellular stream implicated in object recognition (Zeki & Shipp, 1988).”

Other points

In the abstract and throughout, could you replace ‘feedback’ with ‘backward’; similarly, could you replace ‘feedforward’ with ‘forward’? Much 20th-century writing in the visual neuroscience confuses the anatomical designations of forward and backward with the functional designation as feedforward and feedback under predictive coding. In other words, it is the forward prediction errors that are providing the functional feedback.

Line 7: you may want to add: “... takes time (with the exception of generalised predictive coding formulations).”

Line 32: could you say: “and even been advocated as a realisation of the free energy principle - a principle that might apply to all self-organising biological systems.”

The free energy principle is a principle, while predictive coding is a particular process theory that might implement the free energy principle. In light of this, could you nuance the end of your conclusion (line 415), perhaps with something like:

“From the perspective of the free energy principle, the imperative to minimise prediction error can be argued from first principles. This follows because the sum of squared prediction error is, effectively, variational free energy and any system that minimises free energy will, by definition, self-organise to its preferred physiological and perceptual states.”

Line 63: to leverage the free energy principle perspective, you could also say here:

“Theoretically, the imperative to minimise complexity and metabolic cost is an inherent part of free energy (i.e. prediction error) minimisation. This follows because the free energy provides a proxy for model evidence - and model evidence is accuracy minus complexity. This means that minimising free energy is effectively the same as self-evidencing (Hohwy, 2016) and both entail a minimisation of complexity or computational costs - and their thermodynamic concomitants.

I hope that these comments help should any revision be required.

Friston, K., Stephan, K., Li, B., & Daunizeau, J. (2010). Generalised Filtering. Mathematical Problems in Engineering, vol., 2010, 621670.

Hohwy, J. (2016). The Self-Evidencing Brain. Noûs, 50(2), 259-285. doi:10.1111/nous.12062

Perrinet, L. U., Adams, R. A., & Friston, K. J. (2014). Active inference, eye movements and oculomotor delays. Biol Cybern, 108(6), 777-801. doi:10.1007/s00422-014-0620-8

Zeki, S., & Shipp, S. (1988). The functional logic of cortical connections. Nature, 335, 311-317.

Reviewer 2

Summary

The authors propose an extended predictive coding framework for processing time-varying stimuli that accounts for processing delays in the neural system. Here, predictions for a future state is made at both feedback and feedforward stages in the hierarchy based on extrapolation mechanisms. This is enabled by exploiting the ‘rate-of-change’ information represented at each stage. It is argued that the model has neural plausibility and can explain multiple perceptual phenomena. The proposed model addresses an important question of dealing with prediction errors for dynamic stimuli like visual motion and the idea of implementing extrapolation at every stage is intriguing. However, some of the core concepts consisting the model are not fully explained, and it is not clear whether the review of the current literature sufficiently supports the key ideas.

Major Concerns

1. It is somewhat difficult to grasp what kind of computation is needed for the model to operate. Extrapolation seems to be an important concept in the proposed model, but what is being extrapolated based on what information is not clear. Can the authors maybe provide a more concrete example and/or better clarify the variables using a simple equation to explain this? Figure 2 seems to illustrate an example where the position is extrapolated. But it is difficult to pinpoint, for example, what variables are available and/or inferred from where. Below concerns are also related to this issue.

2. Similarly, the rate-of-change information, which seems to be a critical part in this computation, is somewhat vague. The authors link it to velocity, but there are some unanswered questions. For example, how is this velocity information acquired? For a system to make a prediction, it seems that the velocity of a moving stimulus needs to be first learned. If this is the case, how is this information learned and over what time scale, especially when the feedforward signals also represent not the current input but rather the predictions (which may also be prone to errors)?

3. Also, the assumption here seems to be that the information about inherent processing delays in different stages should be available at some point in the computation. For a successful temporal alignment, a given unit should be able to predict further in time that correctly accounts for its delay time. This seems to very critical to the model, but it is not well explained how the system would ‘know’ such information (which also seems to be a very difficult job to accomplish).

4. More importantly, the above questions are related to the issue of neural plausibility. In the simplest terms, for instance, the receptive field properties of visual neurons in each hierarchy will vary - how is this implemented in the framework? Will this information be helpful in elaborating the model?

5. A dynamic predictive coding framework has been proposed in the past (Hosoya et al 2005) where the spatio-temporal structure of the receptive field properties adapt to the changing stimulus statistics. This could potentially account for processing time-varying stimuli. Also, there was an attempt to explain the receptive field properties for motion stimuli using a predictive coding mechanism (e.g., Jenekee et al 2006). How is the authors' proposal different from previous studies?

6. Conceptually, it is not clear why the misalignment-the prediction error at each stage-is necessarily bad, or, something that should be minimized. It has been suggested that the representation of error signals in a predictive coding framework increases efficiency in neural coding (e.g., Huang & Rao 2011). The assumption in the current paper seems to be somewhat opposite from it.

Minor points

1. Lines 232-239: the studies mentioned here provide evidence for prediction (that predicted information is represented somewhere in the brain), but is not necessarily related to representing prediction error (which is essential in the predictive coding) or extrapolation at both feedback and feedforward stages? In other words, is predictive coding needed to explain those results? More explanations might be helpful.

2. Line 252: what does it mean that each connection can compensate for its own delay in model C? Further explanations may be helpful.

3. Lines 254-270: can the authors add more explanations on why the model B can't explain the introduced phenomena?

4. Line 273: it will be helpful to see some explanations on how motion is incorporated into the model to interact with position information.

5. Lines 321-334: implications about local time perception seems stretchy unless the importance of local circuitry in the proposed model is better explained.
